# Diagnostic Cerebrospinal Fluid Biomarker in Early and Late Onset Multiple Sclerosis

**DOI:** 10.3390/biomedicines10071629

**Published:** 2022-07-07

**Authors:** Franz Felix Konen, Malte Johannes Hannich, Philipp Schwenkenbecher, Matthias Grothe, Konrad Gag, Konstantin Fritz Jendretzky, Stefan Gingele, Kurt-Wolfram Sühs, Torsten Witte, Thomas Skripuletz, Marie Süße

**Affiliations:** 1 Department of Neurology, Hannover Medical School, 30625 Hannover, Germany; konen.felix@mh-hannover.de (F.F.K.); schwenkenbecher.philipp@mh-hannover.de (P.S.); jendretzky.konstantin@mh-hannover.de (K.F.J.); gingele.stefan@mh-hannover.de (S.G.); suehs.kurt-wolfram@mh-hannover.de (K.-W.S.); 2 Institute of Clinical Chemistry and Laboratory Medicine, University Medicine Greifswald, 17475 Greifswald, Germany; malte.hannich@med.uni-greifswald.de; 3 Department of Neurology, University Medicine Greifswald, 17475 Greifswald, Germany; matthias.grothe@med.uni-greifswald.de (M.G.); konrad.gag@med.uni-greifswald.de (K.G.); marie.suesse@uni-greifswald.de (M.S.); 4 Department of Rheumatology & Immunology, Hannover Medical School, 30625 Hannover, Germany; witte.torsten@mh-hannover.de

**Keywords:** multiple sclerosis, free light chains kappa, cerebrospinal fluid, humoral immune response, immunoglobulins, oligoclonal bands, MRZ reaction, early onset MS, late onset MS, progressive MS

## Abstract

**Background:** The intrathecal humoral response is the characteristic diagnostic finding in the cerebrospinal fluid (CSF) analysis of patients with multiple sclerosis (MS). Although the average age of MS patients increases, little is known about the sensitivity of diagnostic markers in elderly MS patients. **Methods:** In this retrospective two-center study, intrathecal free light chains kappa fraction (FLCk IF) and oligoclonal bands (OCB) were studied in a large cohort of patients with early and late onset relapsing (RMS) and progressive (PMS) MS. Furthermore, the humoral immune profile in CSF was analyzed, including the polyspecific intrathecal immune response measured as the MRZ reaction. **Results:** While the frequency of CSF-specific OCB did not differ between early and late onset RMS and PMS, the sensitivity of positive FLCk IF and absolute FLCk IF values were lower in PMS. The positivity of the MRZ reaction was equally frequent in early and late onset RMS and PMS. PMS patients had higher local IgA concentrations than RMS patients (*p* = 0.0123). **Conclusions:** OCB are slightly superior to FLCk IF in progressive MS in terms of sensitivity for detecting intrathecal immunoglobulin synthesis. The MRZ reaction, as the most specific parameter for MS, is also applicable in patients with late onset and progressive MS.

## 1. Introduction

The detection of an intrathecal humoral immune response is the characteristic finding in the cerebrospinal fluid (CSF) analysis of patients with multiple sclerosis (MS) [1,2]. Since intrathecal immunoglobulin (Ig) G synthesis can be detected in CSF by an oligoclonal band (OCB) analysis in up to 99% of adult MS patients, this biomarker has been included in the diagnostic criteria for MS [1,2,3,4,5]. Free light chains kappa (FLCk) secreted by plasma cells also reflects the intrathecal Ig synthesis and has gained importance in the diagnosis of MS as an easily measured, quantifiable biomarker with similar sensitivity compared to the OCB analysis [4,5,6,7,8]. The intrathecal humoral immune response of MS patients is stable during the disease course, and the frequency of an intrathecal IgG synthesis measured by OCB detection is the same in pediatric and adult MS [1]. However, less attention is paid to the humoral immune response in MS disease at older ages, especially in a MS diagnosis in patients older than 50 years (so-called late onset MS). The diagnosis of late onset MS patients is difficult, because the clinical presentation may be unusual, and thus, they are often misdiagnosed [9,10,11]. The risk of conversion to a progressive disease course with the dominance of neurodegenerative processes compared to the typical inflammatory changes in relapsing-remitting stages increases with age [12,13]. Based on the disease course and disability, a distinction is made between relapsing-remitting (RMS), primary-progressive (PPMS), and secondary-progressive (SPMS) MS [14,15]. Primary-progressive MS accounts for 10% of all MS patients and is more common in elderly and male patients [16,17,18]. Due to a greater awareness of MS in elderly patients and greater availability of MRI diagnostics, the number of patients older than 50 years first diagnosed with MS is increasing [11,12]. It also has been shown that immunomodulatory or immunosuppressive MS therapies may not be as effective in elderly MS patients [11,19]. Therefore, there is a need for better understanding the immune pathogenesis in elderly MS patients. In the present study, we therefore aim to answer two questions: (I) Are the current diagnostic CSF biomarkers suitable for the detection of an intrathecal humoral immune response in elderly MS patients? (II) Can the differences in these biomarkers provide evidence of an altered intrathecal humoral immune response in late onset MS and different disease courses? To answer these, a detailed humoral intrathecal immune profile consisting of the locally synthesized Ig and FLCk concentrations, the intrathecal fraction of FLCk, the OCB types, and the polyspecific intrathecal immune response, measured as a MRZ reaction (measles, rubella, and zoster CSF/serum antibody index), was studied in a large cohort of MS patients with early onset and late onset MS, with particular reference to different MS courses.

## 2. Materials and Methods

### 2.1. Patients

This retrospective, two-center study compromises a total of 250 patients, including 183 patients with RMS, 55 with PPMS, and 12 with SPMS. The latter are grouped under progressive MS (PMS) in the following analysis (*n* = 67) [20]. The patients were selected according to their diagnosis. Disease progression was assumed to be clinical progression independent of relapses for at least one year with at least one point in Kurtzke’s expanded disability status scale (EDSS) assessment [15,20,21]. Of the 250 patients, 186 were classified as having early onset MS, because the first clinical events suggestive of MS occurred before the age of 50 years, while 64 patients were classified as having late onset MS, with the first clinical event after the age of 50 years [9]. Patients presented to the Department of Neurology at Hannover Medical School (MHH) or the Department of Neurology at University Medicine Greifswald (UMG) between 2005 and 2021, and a MS diagnosis according to the revised 2017 McDonald criteria have been included in the analysis [3]. Additional information on patient characteristics, basic CSF analytic results, and clinical data is described in Table 1.

### 2.2. Analytical Procedures

Paired CSF and serum samples were analyzed in the Neurochemistry Laboratory of the Department of Neurology of the MHH and in the Interdisciplinary CSF Laboratory of the UMG, according to routine diagnostic procedures. Fuchs-Rosenthal counting chambers were used to manually determine the cell count in the CSF. Kinetic nephelometry (Beckman Coulter IMMAGE, Brea, CA, USA (MHH); BN ProSpec, Siemens Healthcare Diagnostics Products GmbH, Marburg, Germany (UMG)) was used to measure the albumin, IgG, IgM, and IgA concentrations in CSF and serum samples. Reiber´s quotient diagrams were used to estimate the intrathecal synthesized fraction of IgG, IgA, and IgM [22]. Isoelectric focusing in polyacrylamide gels (EDC, Tübingen, Germany), followed by silver staining (MHH) (*n* = 172/250 (69%), early onset RMS = 107/160 (67%); late onset RMS = 8/23 (35%); early onset PMS = 23/26 (88%); late onset PMS = 34/41 (83%)) or isoelectric focusing with a semi-automated agarose electrophoresis system (Hydragel 9 CSF, Hydrasys 2Scan, Sebia GmbH, Fulda, Germany) (UMG) (*n* = 78/250 (31%), early onset RMS = 53/160 (33%); late onset RMS = 15/23 (65%); early onset PMS = 3/26 (12%); late onset PMS = 7/41 (17%)) was used to detect CSF-specific OCB [23]. The following OCB patterns were distinguished: no bands in CSF and serum (type 1), bands in CSF-only (type 2), bands in CSF and additional identical bands in serum and CSF (type 3), and identical bands in serum and CSF (type 4) [24]. As an indicator of renal function, the estimated glomerular filtration rate (eGFR) was calculated using the CKD-EPI creatinine equation [25].

### 2.3. FLCk Determination

To determinate FLCk concentrations in CSF and serum samples, a nephelometric assay (N Latex FLC kappa Kit; Siemens Healthcare Diagnostics Products GmbH, Erlangen, Germany) was used in both centers according to the manufacturer’s instructions on a BN ProSpec analyzer (Siemens Healthcare Diagnostics Products GmbH, Erlangen, Germany). The CSF predilution was set to 1:2, the serum predilution was set to 1:100, and the lower limit of quantification of the assay was set to 0.034 mg/L. The hyperbolic reference range and the amount of intrathecally synthesized FLCk (FLCk IF) and IgG, IgA, and IgM were calculated according to the formulas described by Reiber et al. (discrimination line: Q_lim_ (FLCk) = (3.27(Q_Alb_^2^ + 33)^0.5^ − 8.2) × 10^−3^; $Q_{\lim}\;\left(\mathrm{IgG}\right)=0.93\times \sqrt{{\left(\mathrm{QAlb}\right)}^{2}+6\times 10^{-6}}-1.7\times 10^{-3}$; $Q_{\lim}\;\left(\mathrm{IgA}\right)=0.77\times \sqrt{{\left(\mathrm{QAlb}\right)}^{2}+23\times 10^{-6}}-3.1\times 10^{-3}$; $Q_{\lim}\;\left(\mathrm{IgM}\right)=0.67\times \sqrt{{\left(\mathrm{QAlb}\right)}^{2}+120\times 10^{-6}}-7.1\times 10^{-3}$; and reference range: Q_mean_ (FLCk/IgG/IgA/IgM) ± 3 CV) [4]. For statistical comparisons, the local concentration of FLCk, IgG, IgA, and IgM (FLCk/IgG/IgA/IgM_loc_) was calculated as follows: FLCk/IgG/A/M_loc_ = (Q_FLCk/IgG/IgA/IgM_ − Q_mean FLCk/IgG/IgA/IgM_) × FLCk/IgG/IgA/IgM serum (mg/L) [4,7].

### 2.4. Polyspecific Immune Response

The polyspecific immune response, known as the MRZ-reaction, was considered partially positive if at least two out of three virus-specific antibody indices (AI) against measles, rubella, and varicella zoster (VZV) viruses were elevated. Virus-specific AI were calculated using the following formula: (CSF virus-IgG/serum virus-IgG)/(CSF IgG total/serum IgG total) (positive: AI > 1.4) [26,27]. Viral IgG was measured in the CSF and serum using ELISA kits from Virion/Serion (Würzburg, Germany) (MHH). A polyclonal rabbit anti-human IgG-HRP from Agilent (Santa Clara, CA, USA) was used as the detection antibody (MHH). For UMG samples, an in-house ELISA method was used. Virion/Serion (Serion Elisa classic, Würzburg, Germany) microtiter plates and antibodies from SIGMA (P0214), conjugated with alkaline phosphatase, were used. Concentrations of viral IgG in the CSF and serum were determined using the standard curve [26,27].

### 2.5. Statistical Analysis

Statistical analysis and graphical processing were performed using GraphPad Prism (La Jolla, CA, USA; version 5.02), SPSS 25.0 (IBM Co., Armonk, NY, USA), and RStudio (R version 3.5.1 2018-07-02). The statistical significance level was set at 5%. The D’Agostino & Pearson omnibus normality test was applied to assess the normal distribution. Mean, median, minimum (min), and maximum (max) were used to describe the data. The Mann–Whitney *U* Test was used to analyze the independent values. The Kruskal–Wallis test and the Friedman test with Dunn’s Multiple Comparison post hoc test were used to compare the groups. Contingency table analyses were performed using chi-square and Fisher´s exact test. Significant correlations were assessed with Spearman’s r (nonparametric distributed values) and Pearson’s r (Gaussian distributed values). A multiple regression analysis was performed to investigate the factors influencing the humoral CSF parameters (magnetic resonance imaging (MRI) gadolinium enhancement, EDSS, disease course, and early or late onset). Bonferroni correction was used for the post hoc analyses.

## 3. Results

Demographic data and basic CSF parameters of the included patient samples are shown in Table 1. None of the included patients (*n* = 250) were receiving effective disease-modifying therapy (DMT) at the time of a lumbar puncture. In early onset MS patients, an RMS disease course was more frequent than a PMS course, whereas, in late onset MS patients, a PMS disease course was more frequently observed. In 90% (*n* = 225) of the patients, an MRI of the brain was available at the time of CSF sampling. Gadolinium enhancement was present in 112 out of 225 patients (50%). Patients with RMS had a significantly higher proportion of gadolinium-enhancing inflammatory MRI lesions than patients with PMS independent of the age of onset (*p* < 0.0001). The median EDSS score as a measure of MS-associated disability was higher in patients with PMS than in patients with RMS independent of the age of onset (*p* < 0.0001).

### 3.1. Different OCB Pattern in Progressive versus Relapsing MS

**Figure 1:**
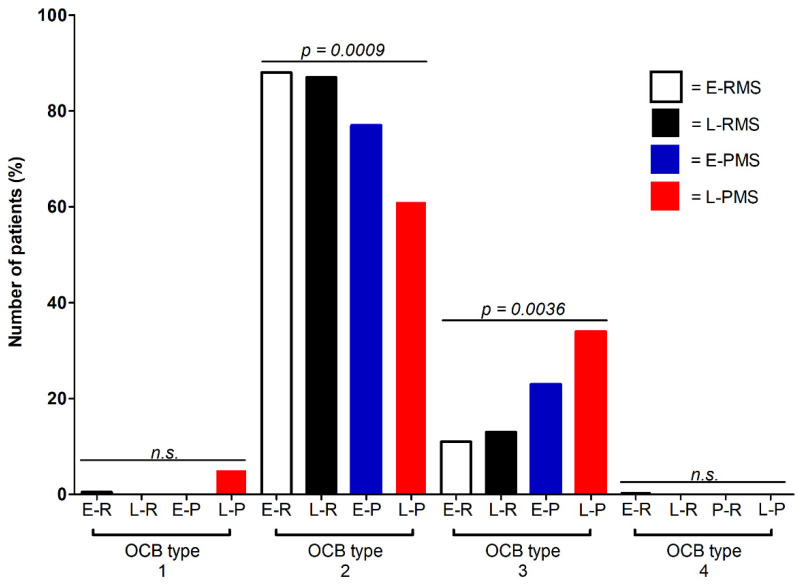
Oligoclonal band (OCB) pattern in multiple sclerosis. E-R/E-RMS = early onset relapsing multiple sclerosis, L-R/L-RMS = late onset relapsing multiple sclerosis, E-P/E-PMS = early onset progressive multiple sclerosis, L-P/L-PMS = late onset progressive multiple sclerosis, and n.s. = not statistically significant. Overall, the *p*-values for OCB types 2 and 3 are statistically significant. For OCB type 2, the comparison of early and late onset RMS and early and late onset PMS was not statistically significant (RMS *p* = 1, PMS: 0.1956). Comparison between the early onset of RMS and PMS, as well as between late onset RMS and PMS, was statistically significant (early onset MS: *p* = 0.2168; late onset MS: *p* = 0.0449). For OCB type 3, comparison between early and late onset RMS and early and late onset PMS was not statistically significant (RMS *p* = 0.7320, PMS 0.4168). Comparison between the early onset of RMS and PMS, as well as late onset RMS and PMS, was statistically significant (early onset MS: *p* = 0.1137; late onset MS: *p* = 0.0466).

The OCB analysis showed a diagnostic sensitivity of 99% in early and 100% in late onset RMS and 95% in late and 100% in early onset PMS (*p* = 0.2934). Samples from patients with early onset and late onset RMS showed type 2 OCB patterns more frequently than patients with early and late onset PMS (overall *p* = 0.0009) (Figure 1). OCB type 3, on the other hand, was more frequent in patients with PMS compared to patients with RMS (overall *p* = 0.0036). When comparing OCB types 2 and 3 between early and late onset RMS, as well as between early and late onset PMS patients, no statistically significant differences became apparent (OCB 2: RMS *p* = 1, PMS: 0.1956; OCB 3: RMS *p* = 0.7320, PMS 0.4168). In contrast, a comparison of OCB types 2 and 3 between late onset RMS and late onset PMS patients revealed a significantly lower frequency of OCB type 2 and a higher frequency of OCB type 3 in PMS patients (OCB type 2: *p* = 0.0449; OCB type 3: *p* = 0.0466).

### 3.2. Equal Sensitivity of the MRZ Reaction in Early and Late Onset RMS and PMS

**Figure 2:**
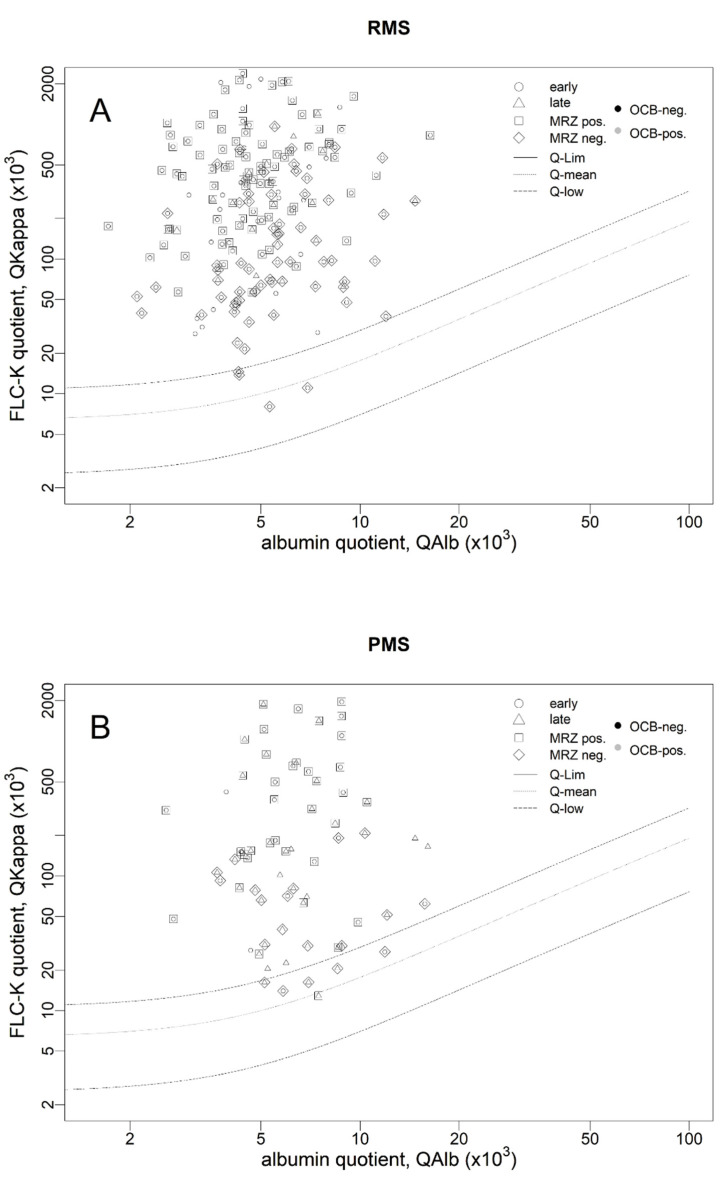
FLCk quotients of the RMS cohort (**A**) and PMS cohort (**B**) in double logarithmic free light chain kappa (FLCk) quotient diagrams. OCB positivity is represented by black and negativity by grey coloration. Circles represent early onset, triangles late onset MS squares represent samples with MRZ positivity, rhombus MRZ negativity. (**A**) In RMS patients, 98% of the QFLCk values are above the Q_FLCk_ (lim) reference range in early onset and in 100% in late onset RMS. (**B**) In PMS patients, 90% of the QFLCk values are above the Q_FLCk_ (lim) reference range in early onset and in 92% in late onset PMS. IF = intrathecal fraction, FLCk = free light chains kappa, MS = multiple sclerosis, RMS = relapsing MS, PMS = progressive MS, OCB = oligoclonal bands, and MRZ = measles, rubella, and zoster reaction.

The polyspecific immune response (“MRZ reaction”) as the most specific parameter for MS was equally frequent in all investigated patient cohorts (*p* = 0.4950): RMS with early onset (80/136, 59%) and late onset (12/20, 60%) and PMS with early onset (18/24, 75%) and late onset (19/33, 58%) (Figure 2A,B).

### 3.3. Lower Sensitivity of the FLCk IF in Progressive MS

**Figure 3:**
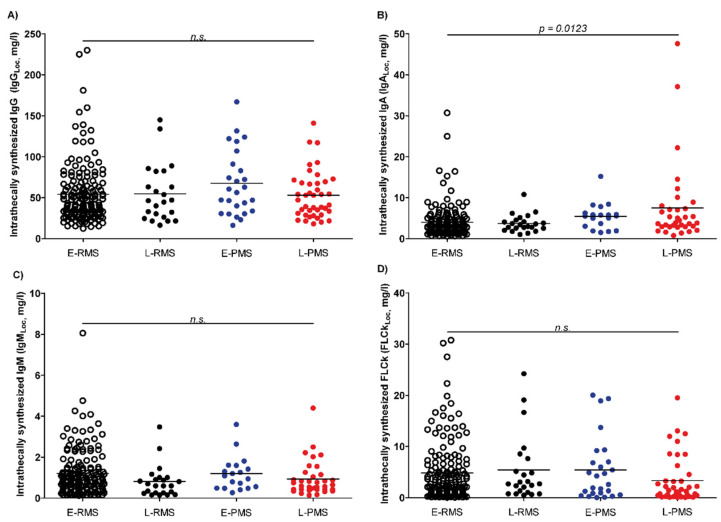
Levels of intrathecally synthesized IgG (**A**), IgA (**B**), IgM (**C**) and free light chains kappa; FLCk (**D**) given as local concentrations. Loc = Local concentration, E-RMS = early onset relapsing multiple sclerosis, L-RMS = late onset relapsing multiple sclerosis, E-PMS = early onset progressive multiple sclerosis, L-PMS = late onset progressive multiple sclerosis, and n.s. = not statistically significant. Overall, the *p*-value in (**B**) is statistically significant. Comparison between early onset and late onset RMS and PMS was not statistically significant (early versus late RMS: *p* = 0.4769; early versus late PMS: 0.6638). Comparison of early onset RMS and PMS, as well as late onset RMS and PMS, was statistically significant for early, as well as late, onset MS (early onset MS: *p* = 0.0073; late onset MS: *p* = 0.0077).

As shown in Figure 2A and Table 1, the FLCk IF is applicable to the first lumbar puncture in patients with RMS with a sensitivity of 98% and 100% (early and late onset) and with a lower sensitivity of 92% and 90% (early and late onset) in patients with PMS (Figure 2B). Frequencies of FLCk IF positivity were not statistically different between early and late onset MS patients independent of the disease course (RMS early versus late: *p* = 1; PMS early versus late: *p* = 1). Furthermore, frequencies of FLCk IF positivity were not statistically different between PMS and RMS in patients with early as well as late onset MS (early onset RMS and early onset PMS: *p* = 0.1979; late onset RMS and late onset PMS: *p* = 0.2876). Whereas there was no significant difference in FLCk levels (FLCk_Loc_) between early and late onset RMS as well as early and late onset PMS (*p* = 0.0940, Figure 3D), patients with PMS have lower absolute FLCk IF values than RMS patients independent of the age of onset (*p* = 0.0009, age-corrected *p* = 0.0153). To shed more light on these results, we have included the following analyses: absolute FLCk values in sera were significantly higher in MS patients with late onset independent of the disease course (*p* < 0.0001, age-corrected *p* = 0.0263). Absolute CSF FLCk concentrations were not significantly different between different MS disease courses (*p* = 0.1339).

No correlation between MRI gadolinium enhancement or EDSS and local FLCk concentrations was demonstrated (*p* = 0.751, *p* = 0.917, respectively). The renal function estimated by eGFR was significantly lower in patients with late onset MS compared with early onset MS patients (*p* < 0.0001, age-corrected *p* < 0.0001), independent of the disease course.

### 3.4. Changes in Locally Synthesized IgA in Progressive MS

Intrathecally synthesized IgG levels given as local concentrations (IgG_Loc_) were not significantly different between cohorts, as shown in Figure 3A (*p* = 0.2277). Looking at the intrathecally synthesized IgA and M levels given as local concentrations (IgA_Loc_, IgM_Loc_) shows that no significant differences between early and late onset RMS and early and late PMS were found for local IgM concentrations (*p* = 0.0845) but that PMS patients have higher local IgA concentrations in the CSF than RMS patients, independent of the age of onset (overall *p* = 0.0123) (Figure 3B,C). When comparing IgA_Loc_ of early and late onset RMS, as well as early and late onset PMS patients, no statistically significant differences became apparent (early versus late onset RMS: *p* = 0.4769; early versus late onset PMS: 0.6638). In contrast, a comparison between IgA_Loc_ of early onset RMS and early onset PMS, as well as between late onset RMS and late onset PMS patients, revealed significantly higher concentrations in PMS patients (early onset MS: *p* = 0.0073; late onset MS: *p* = 0.0077).

## 4. Discussion

In the present study, we were able to show changes in the diagnostic CSF biomarkers in the form of a lower frequency of an intrathecal fraction of FLCk in MS patients with a progressive disease course, as well as changes in the humoral immune profile in the form of higher local IgA concentrations in patients with progressive MS.

Immunological changes in the disease course of MS associated with ageing include B-cell immunosenescence [28,29]. This term covers the reduction and functional alterations of the naïve B-cell population, as well as a decrease in the clonal expansion capacity of memory cells, antibody levels, and a decrease in antibody specificity [19,28,29]. Based on this, it could be hypothesized that the humoral immune response in the CNS also changes, which would have implications for the diagnosis of MS in older ages.

An important finding of the present study is that the detection of an intrathecal fraction of FLCk as a diagnostic biomarker is less frequent in PMS than in RMS patients independent of the age of onset, although this difference is not statistically significant. This should be taken into account when considering the analysis of FLCk in the diagnosis of progressive MS. In comparison, the analysis of OCB as the previous reference standard is more sensitive for detecting intrathecal Ig synthesis in these patient cohorts. One reason for the slightly lower diagnostic sensitivity of the intrathecal fraction of FLCk compared with OCB in our study may be the method of OCB determination. All FLCk negative samples revealed pathological OCB results in the silver staining method after isoelectric focusing on polyacrylamide gels. Studies using silver staining to detect OCB showed a better performance with higher rates of OCB-positive patients than other methods of OCB determination, even if the cause of this has not been conclusively clarified [30]. Therefore, we recommend that the OCB detection is performed by silver staining if a clinical suspicion of MS and negative FLCk levels are present. Another known risk factor for a false negative intrathecal fraction of FLCk values is pathologic renal function parameters and elevated serum FLCk levels [31]. Although the patients described here with negative FLCk IF did not have pathologic renal function parameters or elevated FLCk serum levels, the absolutely lower FLCk IF values in late onset MS patients suggest that the risk for false-negative FLCk IF values increases with age [31]. A recent study suggested that the age-related impairment of renal function in elderly patients may result in decreased urinary excretion of FLCk and subsequently higher serum concentrations [31]. Consistent with these observations, the patients with late onset RMS and the PMS patients in our study had significantly lower renal function, as measured by eGFR (Table 1). Therefore, our results suggest that the intrathecal fraction of FLCk should be interpreted carefully as a diagnostic parameter in PMS patients with symptoms suggestive of the first clinical event of MS, especially when the renal function is impaired or serum FLCk concentrations are elevated due to increased synthesis [4,31]. In these cases, an OCB analysis should preferably be performed with silver staining to exclude “false” negative FLCk IF results.

Several studies have proposed FLCk as a prognostic biomarker in MS, as correlations between FLCk concentrations in CSF, FLCk indices, or the intrathecal fraction of FLCk and disease progression, according to EDSS, have been reported [32,33]. However, the major limitations of these studies are that few patients with high EDSS scores were included, and neither renal function impairment nor higher serum FLCk values were considered [31,32,33,34]. In addition, the results in progressive MS patients have not been reported in detail [32,33,35,36,37,38,39]. In the present study, intrathecally synthesized FLCk described as a local concentration (FLCk_Loc_) showed no significant correlation with the EDSS scores. In line with these considerations, no correlations between FLCk concentrations or FLCk indices and EDSS were observed in various other studies [40,41,42,43].

So far, there is limited evidence of IgA in different types of MS. However, Abdelhak et al. reported elevated IgA indices in almost 25% of PPMS patients studied [44]. Since a negative correlation was observed between intrathecally produced IgA and the progression rate, a possible protective role of intrathecally produced IgA was suggested [44]. In the present study, IgA_Loc_ in CSF was significantly higher in PMS compared to RMS patients, suggesting a relationship between the relapse rate or disease activity and low CSF IgA_Loc_. However, these findings need further elucidation.

Age-related effects have to be considered not only in the FLCk analysis but also in the analysis of different OCB types. A different distribution of OCB patterns in RMS, SPMS, and PPMS patients has been described previously, with a preponderance of a type 2 pattern in all MS types and a higher prevalence of types 3 and 4 patterns in chronic progressive MS patients [18,45,46]. We were able to replicate this observation. In contrast to the study by Villar et al., which found a dominance of the type 3 OCB pattern in PPMS patients (64% of all included patients), most other studies concluded that a type 2 OCB pattern is predominant in chronic progressive MS patients, as well as in RMS patients [18,45,46,47]. The exceptionally high percentage of a type 3 OCB pattern in PPMS patients reported by Villar et al. points to possible pre-analytic or methodical causes for these findings [18,45]. The high frequency of type 3 OCB patterns in chronic progressive MS and late onset MS may be due to the higher likelihood of systemic infections and blood–CSF barrier dysfunction in older patients in general [18,47,48]. Thus, this phenomenon is most likely age-related and does not reflect a disease-specific mechanism.

Since infectious diseases are more common than chronic autoimmune diseases in the elderly, the MRZ reaction has a great importance for the differential diagnosis of MS in elderly patients. In contrast to the determination of OCB, which are completely nonspecific for MS and only indicate intrathecal nonspecific IgG synthesis, the MRZ response is the most specific parameter for chronic autoimmune CNS diseases currently available [49]. It is the most specific biomarker for the diagnosis of MS, so that equal frequencies of polyspecific immune responses would have been expected in all MS patients of different ages in the present study [50,51]. Furthermore, in the study of Hottenrott et al., PPMS and RMS patients had the same frequency of MRZ positivity, so that the present results are consistent with the literature [51].

### Limitations

It is clear that the humoral intrathecal immune response alone can provide little information about complex pathophysiological immunological processes. Thus, this retrospective analysis cannot rely on the immunophenotyping of B and T cells, which could provide additional information in this regard. As the focus of the study was exclusively on the application of the biomarkers in multiple sclerosis, no statement can be made on the specificity of the diagnostic markers mentioned. To assess the changes in the immune response with age, intraindividual longitudinal analyses would certainly be desirable. Due to the necessary time interval of several intraindividual lumbar punctures to a CSF analysis of several decades to assess the age effect, we conducted this cross-sectional study instead.

## 5. Conclusions

The intrathecal humoral immune response, reflected in the CSF-specific OCB and MRZ response, remains largely stable when comparing different disease courses of MS and the age of diagnosis. This is of utmost importance for the diagnosis of MS in elderly patients, as the current diagnostic criteria use an OCB analysis as a surrogate for the dissemination in time when MRI criteria or a clinical disease course are not sufficient [3]. FLCk IF can also be used with high sensitivity in patients with late onset MS and PMS but is slightly inferior to previous markers in terms of diagnostic sensitivity in these patients.

## Figures and Tables

**Table 1:** Demographic and clinical data.

	RMS, Early Onset (*n* = 160)	RMS, Late Onset (*n* = 23)	PMS, Early Onset (*n* = 26)	PMS, Late Onset (*n* = 41)
Age (years), median (min–max)	31 (18–49)	57 (51–73)	43.5 (22–49)	58 (44–74)
Females/males ratio	2.3	3.6	1.6	3
EDSS, median (min–max)	2 (0–4.5)	2 (0–3.5)	3 (0–7.5)	4 (2–7.5)
Gadolinium-enhancing inflammatory MRI lesions, *n*/available MRI (%)	93/154 (60%)	12/23 (52%)	4/19 (21%)	3/29 (10%)
Cell count (per µL CSF), mean (min–max)	10 (0–96)	5 (1–19)	5 (0–20)	5 (0–31)
CSF-specific oligoclonal bands, *n* (%)	158 (99%)	23 (100%)	26 (100%)	39 (95%)
FLCk concentration in serum (mg/L), mean (min–max)	11 (0.6–33)	13 (4–25)	13 (7–29)	15 (7–32)
FLCk concentration in CSF (mg/L), mean (min–max)	5 (0.09–31)	6 (0.55–24)	6 (0.15–20)	4 (0.13–20)
Intrathecal fraction of FLCk (FLCk IF) according to Reiber’s diagram, *n* (%)	156 (98%)	23 (100%)	24 (92%)	37 (90%)
eGFR (mL/min/1.73 m^2^), mean (min–max)	110 (66–142)	89 (68–111)	101 (74–130)	87 (57–116)

CSF = cerebrospinal fluid, FLCk = free light chains kappa, IF = intrathecal fraction, EDSS = expanded disability status scale, eGFR = estimated glomerular filtration rate, and MRI = magnet resonance imaging.

## Data Availability

The datasets used and/or analyzed during the current study are available from the corresponding author upon reasonable request.

## References

[1] Reiber H., Teut M., Pohl D., Rostasy K.M., Hanefeld F. (2009). Paediatric and adult multiple sclerosis: Age-related differences and time course of the neuroimmunological response in cerebrospinal fluid. Mult. Scler..

[2] Reich D.S., Lucchinetti C.F., Calabresi P.A. (2018). Multiple Sclerosis. N. Engl. J. Med..

[3] Thompson A.J., Banwell B.L., Barkhof F., Carroll W.M., Coetzee T., Comi G., Correale J., Fazekas F., Filippi M., Freedman M.S. (2018). Diagnosis of multiple sclerosis: 2017 revisions of the McDonald criteria. Lancet Neurol..

[4] Reiber H., Zeman D., Kušnierová P., Mundwiler E., Bernasconi L. (2019). Diagnostic relevance of free light chains in cerebrospinal fluid—The hyperbolic reference range for reliable data interpretation in quotient diagrams. Clin. Chim. Acta.

[5] Schwenkenbecher P., Konen F.F., Wurster U., Witte T., Gingele S., Sühs K.W., Stangel M., Skripuletz T. (2019). Reiber’s Diagram for Kappa Free Light Chains: The New Standard for Assessing Intrathecal Synthesis?. Diagnostics.

[6] Süße M., Feistner F., Grothe M., Nauck M., Dressel A., Hannich M.J. (2020). Free light chains kappa can differentiate between myelitis and noninflammatory myelopathy. Neurol. Neuroimmunol. Neuroinflamm..

[7] Süße M., Reiber H., Grothe M., Petersmann A., Nauck M., Dressel A., Hannich M.J. (2020). Free light chain kappa and the polyspecific immune response in MS and CIS—Application of the hyperbolic reference range for most reliable data interpretation. J. Neuroimmunol..

[8] Schwenkenbecher P., Konen F.F., Wurster U., Jendretzky K.F., Gingele S., Sühs K.W., Pul R., Witte T., Stangel M., Skripuletz T. (2018). The Persisting Significance of Oligoclonal Bands in the Dawning Era of Kappa Free Light Chains for the Diagnosis of Multiple Sclerosis. Int. J. Mol. Sci..

[9] Kis B., Rumberg B., Berlit P. (2008). Clinical characteristics of patients with late-onset multiple sclerosis. J. Neurol..

[10] Delalande S., De Seze J., Ferriby D., Stojkovic T., Vermersch P. (2002). Sclérose en plaques de début tardif [Late onset multiple sclerosis]. Rev. Neurol..

[11] Awad A., Stüve O. (2010). Multiple sclerosis in the elderly patient. Drugs Aging.

[12] Sanai S.A., Saini V., Benedict R.H., Zivadinov R., Teter B.E., Ramanathan M., Weinstock-Guttman B. (2016). Aging and multiple sclerosis. Mult. Scler..

[13] Vaughn C.B., Jakimovski D., Kavak K.S., Ramanathan M., Benedict R.H.B., Zivadinov R., Weinstock-Guttman B. (2019). Epidemiology and treatment of multiple sclerosis in elderly populations. Nat. Rev. Neurol..

[14] Brownlee W.J., Hardy T.A., Fazekas F., Miller D.H. (2017). Diagnosis of multiple sclerosis: Progress and challenges. Lancet.

[15] Lublin F.D., Reingold S.C., Cohen J.A., Cutter G.R., Sørensen P.S., Thompson A.J., Wolinsky J.S., Balcer L.J., Banwell B., Barkhof F. (2014). Defining the clinical course of multiple sclerosis: The 2013 revisions. Neurology.

[16] Noseworthy J.H., Lucchinetti C., Rodriguez M., Weinshenker B.G. (2000). Multiple sclerosis. N. Engl. J. Med..

[17] Wolinsky J.S., PROMiSe Study Group (2003). The diagnosis of primary progressive multiple sclerosis. J. Neurol. Sci..

[18] Villar L.M., Masterman T., Casanova B., Gómez-Rial J., Espiño M., Sádaba M.C., González-Porqué P., Coret F., Alvarez-Cermeño J.C. (2009). CSF oligoclonal band patterns reveal disease heterogeneity in multiple sclerosis. J. Neuroimmunol..

[19] Schweitzer F., Laurent S., Fink G.R., Barnett M.H., Reddel S., Hartung H.P., Warnke C. (2019). Age and the risks of high-efficacy disease modifying drugs in multiple sclerosis. Curr. Opin. Neurol..

[20] Meca-Lallana V., Berenguer-Ruiz L., Carreres-Polo J., Eichau-Madueño S., Ferrer-Lozano J., Forero L., Higueras Y., Téllez Lara N., Vidal-Jordana A., Pérez-Miralles F.C. (2021). Deciphering Multiple Sclerosis Progression. Front. Neurol..

[21] Kurtzke J.F. (1983). Rating neurologic impairment in multiple sclerosis: An expanded disability status scale (EDSS). Neurology.

[22] Reiber H. (2003). Proteins in cerebrospinal fluid and blood: Barriers, CSF flow rate and source-related dynamics. Restor. Neurol. Neurosci..

[23] Andersson M., Alvarez-Cermeno J., Bernardi G., Cogato I., Fredman P., Frederiksen J. (1994). Cerebrospinal fluid in the diagnosis of multiple sclerosis: A consensus report. J. Neurol. Neurosurg. Psychiatry.

[24] Freedman M.S., Thompson E.J., Deisenhammer F., Giovannoni G., Grimsley G., Keir G., Ohman S., Racke M.K., Sharief M., Sindic C.J. (2005). Recommended standard of cerebrospinal fluid analysis in the diagnosis of multiple sclerosis: A consensus statement. Arch. Neurol..

[25] Levey A.S., Stevens L.A., Schmid C.H., Zhang Y.L., Castro A.F., Feldman H.I., Kusek J.W., Eggers P., Van Lente F., Greene T. (2009). CKD-EPI (Chronic Kidney Disease Epidemiology Collaboration). A new equation to estimate glomerular filtration rate. Ann. Intern. Med..

[26] Reiber H., Lange P. (1991). Quantification of virus-specific antibodies in cerebrospinal fluid and serum: Sensitive and specific detection of antibody synthesis in brain. Clin. Chem..

[27] Reiber H., Peter J.B. (2001). Cerebrospinal fluid analysis: Disease-related data patterns and evaluation programs. J. Neurol. Sci..

[28] Buffa S., Bulati M., Pellicanò M., Dunn-Walters D.K., Wu Y.C., Candore G., Vitello S., Caruso C., Colonna-Romano G. (2011). B cell immunosenescence: Different features of naive and memory B cells in elderly. Biogerontology.

[29] Grebenciucova E., Berger J.R. (2017). Immunosenescence: The Role of Aging in the Predisposition to Neuro-Infectious Complications Arising from the Treatment of Multiple Sclerosis. Curr. Neurol. Neurosci. Rep..

[30] Konen F.F., Schwenkenbecher P., Jendretzky K.F., Gingele S., Sühs K.W., Tumani H., Süße M., Skripuletz T. (2021). The Increasing Role of Kappa Free Light Chains in the Diagnosis of Multiple Sclerosis. Cells.

[31] Konen F.F., Schwenkenbecher P., Wurster U., Jendretzky K.F., Möhn N., Gingele S., Sühs K.W., Hannich M.J., Grothe M., Witte T. (2021). The Influence of Renal Function Impairment on Kappa Free Light Chains in Cerebrospinal Fluid. J. Cent. Nerv. Syst. Dis..

[32] Rathbone E., Durant L., Kinsella J., Parker A.R., Hassan-Smith G., Douglas M.R., Curnow S.J. (2018). Cerebrospinal fluid immunoglobulin light chain ratios predict disease progression in multiple sclerosis. J. Neurol. Neurosurg. Psychiatry.

[33] Vecchio D., Bellomo G., Serino R., Virgilio E., Lamonaca M., Dianzani U., Cantello R., Comi C., Crespi I. (2020). Intrathecal kappa free light chains as markers for multiple sclerosis. Sci. Rep..

[34] Hannich M.J., Dressel A., Budde K., Petersmann A., Nauck M., Süße M. (2021). Kappa Free Light Chains in the Context of Blood Contamination, and Other IgA- and IgM-Related Cerebrospinal Fluid Disease Pattern. Cells.

[35] Agnello L., Lo Sasso B., Salemi G., Altavilla P., Pappalardo E.M., Caldarella R., Meli F., Scazzone C., Bivona G., Ciaccio M. (2020). Clinical Use of κ Free Light Chains Index as a Screening Test for Multiple Sclerosis. Lab. Med..

[36] Cavalla P., Caropreso P., Limoncelli S., Bosa C., Pasanisi M.B., Schillaci V., Alteno A., Costantini G., Giordana M.T., Mengozzi G. (2020). Kappa free light chains index in the differential diagnosis of Multiple Sclerosis from Neuromyelitis optica spectrum disorders and other immune-mediated central nervous system disorders. J. Neuroimmunol..

[37] Desplat-Jégo S., Feuillet L., Pelletier J., Bernard D., Chérif A.A., Boucraut J. (2005). Quantification of immunoglobulin free light chains in cerebrospinal fluid by nephelometry. J. Clin. Immunol..

[38] Hassan-Smith G., Durant L., Tsentemeidou A., Assi L.K., Faint J.M., Kalra S., Douglas M.R., Curnow S.J. (2014). High sensitivity and specificity of elevated cerebrospinal fluid kappa free light chains in suspected multiple sclerosis. J. Neuroimmunol..

[39] Leurs C.E., Twaalfhoven H., Lissenberg-Witte B.I., van Pesch V., Dujmovic I., Drulovic J., Castellazzi M., Bellini T., Pugliatti M., Kuhle J. (2020). Kappa free light chains is a valid tool in the diagnostics of MS: A large multicenter study. Mult. Scler..

[40] Rosenstein I., Rasch S., Axelsson M., Novakova L., Blennow K., Zetterberg H., Lycke J. (2021). Kappa free light chain index as a diagnostic biomarker in multiple sclerosis: A real-world investigation. J. Neurochem..

[41] Presslauer S., Milosavljevic D., Brücke T., Bayer P., Hübl W. (2008). Elevated levels of kappa free light chains in CSF support the diagnosis of multiple sclerosis. J. Neurol..

[42] Presslauer S., Milosavljevic D., Huebl W., Parigger S., Schneider-Koch G., Bruecke T. (2014). Kappa free light chains: Diagnostic and prognostic relevance in MS and CIS. PLoS ONE.

[43] Berek K., Bsteh G., Auer M., Di Pauli F., Grams A., Milosavljevic D., Poskaite P., Schnabl C., Wurth S., Zinganell A. (2021). Kappa-Free Light Chains in CSF Predict Early Multiple Sclerosis Disease Activity. Neurol. Neuroimmunol. Neuroinflamm..

[44] Reiber H. (2016). Knowledge-base for interpretation of cerebrospinal fluid data patterns. Essentials in neurology and psychiatry. Arq. Neuropsiquiatr..

[45] Abdelhak A., Hottenrott T., Mayer C., Hintereder G., Zettl U.K., Stich O., Tumani H. (2017). CSF profile in primary progressive multiple sclerosis: Re-exploring the basics. PLoS ONE.

[46] McLean B.N., Zeman A.Z., Barnes D., Thompson E.J. (1993). Patterns of blood-brain barrier impairment and clinical features in multiple sclerosis. J. Neurol. Neurosurg. Psychiatry.

[47] Zeman A.Z., Keir G., Luxton R., Thompson E.J. (1996). Serum oligoclonal IgG is a common and persistent finding in multiple sclerosis, and has a systemic source. QJM.

[48] Pannewitz-Makaj K., Wurster U., Jendretzky K.F., Gingele S., Sühs K.W., Stangel M., Skripuletz T., Schwenkenbecher P. (2020). Evidence of Oligoclonal Bands Does Not Exclude Non-Inflammatory Neurological Diseases. Diagnostics.

[49] Reiber H. (2017). Polyspecific antibodies without persisting antigen in multiple sclerosis, neurolupus and Guillain-Barré syndrome: Immune network connectivity in chronic diseases. Arq. Neuropsiquiatr..

[50] Jarius S., Eichhorn P., Franciotta D., Petereit H.F., Akman-Demir G., Wick M., Wildemann B. (2017). The MRZ reaction as a highly specific marker of multiple sclerosis: Re-evaluation and structured review of the literature. J. Neurol..

[51] Hottenrott T., Dersch R., Berger B., Rauer S., Huzly D., Stich O. (2017). The MRZ reaction in primary progressive multiple sclerosis. Fluids Barriers CNS.

